# Label-Free Quantification of Nanoencapsulated Piperonyl Esters in Cosmetic Hydrogels Using Raman Spectroscopy

**DOI:** 10.3390/pharmaceutics15061571

**Published:** 2023-05-23

**Authors:** Suha Elderderi, Franck Bonnier, Xavier Perse, Hugh J. Byrne, Florent Yvergnaux, Igor Chourpa, Abdalla A. Elbashir, Emilie Munnier

**Affiliations:** 1 EA 6295 Nanomédicaments et Nanosondes, Faculté de Pharmacie, Université de Tours, 31 Avenue Monge, 37200 Tours, France; 2 Department of Pharmaceutical Chemistry, Faculty of Pharmacy, University of Gezira, P.O. Box 20, Wad Madani 21111, Sudan; 3 LVMH Recherche, 185 Avenue de Verdun, 45804 Saint Jean de Braye, France; 4 FOCAS Research Institute, TU Dublin, City Campus, Camden Row, D08 CKP1 Dublin 8, Ireland; 5 Bioeurope Groupe Solabia, Route d’Oulins, 28260 Anet, France; 6 Department of Chemistry, College of Science, King Faisal University, P.O. Box 400, Al-Ahsa 31982, Saudi Arabia; 7 Department of Chemistry, Faculty of Science, University of Khartoum, P.O. Box 321, Khartoum 11115, Sudan

**Keywords:** Raman spectroscopy, label-free quantification, alginate nanocarriers, hydrogels, partial least squares regression

## Abstract

Raman spectroscopy is a well-established technique for the molecular characterisation of samples and does not require extensive pre-analytical processing for complex cosmetic products. As an illustration of its potential, this study investigates the quantitative performance of Raman spectroscopy coupled with partial least squares regression (PLSR) for the analysis of Alginate nanoencapsulated Piperonyl Esters (ANC-PE) incorporated into a hydrogel. A total of 96 ANC-PE samples covering a 0.4% *w*/*w*–8.3% *w*/*w* PE concentration range have been prepared and analysed. Despite the complex formulation of the sample, the spectral features of the PE can be detected and used to quantify the concentrations. Using a leave-K-out cross-validation approach, samples were divided into a training set (*n* = 64) and a test set, samples that were previously unknown to the PLSR model (*n* = 32). The root mean square error of cross-validation (RMSECV) and prediction (RMSEP) was evaluated to be 0.142% (*w*/*w* PE) and 0.148% (*w*/*w* PE), respectively. The accuracy of the prediction model was further evaluated by the percent relative error calculated from the predicted concentration compared to the true value, yielding values of 3.58% for the training set and 3.67% for the test set. The outcome of the analysis demonstrated the analytical power of Raman to obtain label-free, non-destructive quantification of the active cosmetic ingredient, presently PE, in complex formulations, holding promise for future analytical quality control (AQC) applications in the cosmetics industry with rapid and consumable-free analysis.

## 1. Introduction

As the skin is the largest organ of the body, trans-dermal is an obvious route for the administration of active pharmaceutical ingredients for the treatment of pathologies, either local or systemic. The development of optimised skincare products to improve consumer well-being and social interactions through skin appearance has also long been the subject of considerable attention in the cosmetics industry. Skin quality and beauty have become significant concerns of the worldwide population, and they consequently drive ongoing research and innovation in the field to develop more effective formulations for the penetration and permeation of the skin by pharmaceutical or cosmetic ingredients (API/ACI). Commercial cosmetics or products are usually semi-solid formulations, such as gels or creams, and generally include complex mixtures of different compounds, such as gelling or thickening agents, humectant agents and antimicrobial ingredients, which allow the product to be more easily spread on the site of application and also enhance their physicochemical and microbiological stability [1,2,3].

Because of its natural function to act as a barrier and protect the body from exogenous insult, achieving dermal penetration of an active ingredient (AI) is not trivial [4], and various strategies have been devised to increase the efficacies of semi-solid formulations to deliver AI across the stratum corneum (SC), the outmost and most protective layer of the epidermis. The encapsulation of AI in nanocarriers (NCs) has been explored most recently and has been demonstrated to successfully improve the efficacy of the delivery of products applied topically [5] by enhancing their penetration through the stratum corneum, thus improving their absorption by the skin [6,7,8,9]. NCs have also been explored to address problems associated with the formulation process itself, such as poor water solubility, or physicochemical stability of an AI, when mixed with the other formula components [10,11]. Moreover, encapsulation can add the possibility to release the AI into the environment in a controlled fashion, potentially enabling a more targeted delivery [12,13,14]. Among the range of NCs that have been explored, core–shell nano-capsules have been identified as particularly promising candidates for delivery vehicles for ACI and API [15,16,17].

Polysaccharides have attracted increasing attention as the principal ingredients of NCs due to their natural origin [9]. Among the most used polysaccharides, alginates are well studied, as they can produce rigid although porous gels when combined with divalent cations. The alginate nanocarriers used in this study (ANCs) are a type of such core–shell nanocarrier, which have been developed for the encapsulation of lipophilic AI. They are composed of a hydrophobic oily core surrounded by a hydrophilic alginate-based shell. They show no toxicity on skin cells and are stable as aqueous suspensions as well as dispersed in semi-solid forms such as hydrogels [12,18,19]. Such semi-solid formulations have become increasingly complex chemical mixtures, as the process of optimisation often entails an increasingly higher multiplicity of ingredients, thus requiring increasingly sophisticated analytical protocols to quantitatively characterise them. The routine analytical quantity control (AQC) of semi-solid formulations can thus be challenging, especially when they contain AI that has been encapsulated in NCs. Pre-analytical protocols require chemical extraction with organic solvents and are followed by analysis using separative techniques such as high-performance liquid chromatography (HPLC), sometimes coupled to mass spectrometry, to achieve quantification [12,20,21]. Large volumes of solvent are required to perform such semi-solid–liquid extractions and HPLC analysis, and the approach is, furthermore, tedious and time consuming. The extraction protocols can also introduce significant errors in the results if they do not achieve 100% recovery of the AI from the formulation studied. Given the current imperative to develop alternatives compliant with the green analytical chemistry principles [22] to limit the production of chemical waste and to reduce the consumption of consumables in industry and research, more environmentally friendly and solventless approaches are increasingly being considered [23,24].

In 2018, a preliminary study by Miloudi et al. investigated vibrational spectroscopy, i.e., attenuated total reflectance infrared (ATR-IR) spectroscopy, as a cost-effective, label-free and non-destructive analytical tool for the quantification of piperonyl esters (PE), a skin lightening agent, encapsulated in ANCs and dispersed in hydrogels [25]. Indeed, in addition to being promising lightening agents, PE are a good model for this type of study because their chemical structure gives them a characteristic molecular signature, enabling quantitative analysis. A follow-up study by Bonnier et al. further demonstrated that a single drop of cosmetics such as hydrogel products containing ANCs-encapsulated PE analysed by ATR-IR spectroscopy without any pre-analytical requirement, i.e., extraction protocols, enabled the construction of accurate predictive models [26]. Raman spectroscopy is a complementary technique to infrared spectroscopy [27], which also provides specific molecular fingerprints to probe the chemical composition of samples analysed [28]. The technique measures the inelastically scattered radiation (the Raman effect) obtained after the illumination of samples with a monochromatic laser source [29]. Many commercially available Raman devices operate in a microscopic mode, allowing access to molecular information at the micrometric level under confocal conditions [30,31]. While Raman spectroscopy has been extensively studied in research for the mapping or profiling of biological tissues and cells for disease diagnosis and other biomedical applications [32,33,34,35,36,37,38] or even subcellular analysis [39,40,41], it remains a powerful analytical technique. In the field of process analytical technology (PAT), Raman spectroscopy is used to monitor and control chemical and pharmaceutical processes [42,43,44], to predict end points of chemical synthesis reactions [45], and to track polymorphic changes in crystallisation processes [46,47]. Additionally, Raman spectroscopy has been reported as a powerful technique enabling quantitative analysis for a wide range of samples, such as for the determination of water in natural deep eutectic solvents [48,49,50], as a quality control tool for chemotherapeutic solutions [51,52], for the quantification of API in solid dosage forms [53,54,55], screening human body fluids such as serum [56,57] or for investigating the distribution of AI in complex cosmetic dried films to study their homogeneity [58]. While ATR-IR spectroscopy requires the withdrawal of samples, Raman spectroscopy enables in situ analysis of samples, i.e., directly through containers [59,60]. The advantages of this are that it ensures the integrity of samples, prevents cross-contamination, and, importantly, protects workers from exposure to potentially harmful substances [60,61]. 

As a follow-up to the previous studies of Miloudi et al. [25] and Bonnier et al. [26], again using the ANC-PE system as a model, the present study aims to demonstrate the potential of Raman spectroscopy coupled with multivariate data mining protocols, namely partial least squares regression (PLSR), for the quantification of nanoencapsulated piperonyl esters (PE) in cosmetic hydrogels. While the recommended final concentration of this ACI in a commercial cosmetic formulation is commonly between 1 and 2% *w*/*w*, the technique will be applied to samples of systematically varied concentrations over the range of 0.4% *w*/*w* PE–8.3% *w*/*w* PE for the purpose of evaluating the accuracy of the approach for this model system. Ultimately, the aim is to demonstrate the suitability of the techniques for analytical quality control (AQC) applications in the cosmetics industry with rapid and consumable-free analysis.

## 2. Materials and Methods

### 2.1. Preparation and Characterisation of Piperonyl Esters-Loaded Alginate Nanocarriers (ANC-PE)

#### 2.1.1. Reagents

The commercial formulation, marketed as Omegalight^®^, was provided by Bioeurope (Solabia group, Anet, France). It is a liquid skin-lightening agent resulting from the combination of piperonyl alcohol and linoleic acid in the form of an ester dissolved in dicaprylyl ether. It is hereafter referred to as Piperonyl Ester (PE), its chemical name. 

Polyoxyethylene sorbitan monooleate (polysorbate 80, Seppic, Castres, France), sorbitan monooleate (Seppic, Castres, France), Sodium alginates (Sigma-Aldrich, Saint-Quentin-Fallavier, France) and calcium chloride (Fisher Scientific, Illkirch, France) were used to prepare ANC. 

Other compounds used to prepare the semi-solid-form model are sodium carboxymethylcellulose (CMC) as a gelling agent (Fisher Scientific, Illkirch, France), Cosgard^®^ as a preservative (benzyl alcohol and dehydroacetic acid, Aroma zone, Paris France) and glycerol as a humectant agent (Cooper, Melun, France). Water was purified using a Milli-Q system (Millipore Corporation, Bedford, MA, USA)

#### 2.1.2. Preparation of ANC-PE Aqueous Suspension

Alginate nanocarriers loaded with PE (ANC-PE) were prepared following the oil-in-water emulsification and ionic gelation protocol described in detail by Nguyen et al. [12]. A sodium alginate aqueous solution (0.6 g/L) was prepared and filtered through a 0.45 µm nylon filter. To prepare the aqueous phase of the emulsion, polysorbate 80 (0.06 g/L) was added to the filtrate. Then, PE (0.015 g/g) and sorbitan monooleate (0.1 g/g) were mixed to form the oil phase. Finally, the nano-emulsification of the two phases was achieved using an ultrasonic probe (Vibra-cell ultrasonic processor, Sonics, 20 kHz) for 3 min. The addition of an aqueous solution of calcium ions (0.67 g/L) led to the gelation of the surface of the nanocarrier. The final concentration of the active ingredient of the ANC-PE aqueous suspension was 0.165 g/g.

#### 2.1.3. Physicochemical Characterisation of ANC-PE Suspensions 

Dynamic light scattering (DLS), using a Zetasizer instrument (Malvern Panalytical, Malvern, UK), was used to assess the average hydrodynamic diameter and the polydispersity index of ANC-PE. The assays were performed on the samples diluted 1/40 in ultrapure water, with a 633 nm laser source, at 173°. Zeta potentials (ζ) were measured using the same equipment with the detection angle set at 13°. All measurements have been performed in triplicate at 25 °C.

#### 2.1.4. Preparation of Model Cosmetic Gels 

First, a hydrogel containing 3% *w*/*w* CMC, 40% *w*/*w* of glycerol and 2% *w*/*w* of Cosgard^®^ was prepared in ultrapure water. Continuous mechanical stirring (up to 2000 rpm) was applied with a propeller (Turbotest^®^ by VMI Rayneri, montaigu-Vendée, France) until the formation of a viscous gel. 

Second, 12 sets of samples with systematically varying concentrations of PE were independently prepared, and each set contained 8 samples, for a total of 96 samples. For clarity, the list of samples and corresponding concentrations are summarised in Table 1. As examples, sample C1 from SET_01 was prepared by mixing 2.504 g of the hydrogel, 0.121 g of the ANC-PE suspension and 2.383 g of deionised water, while sample C8 from SET 1 was prepared by mixing 2.551 g of the hydrogel and 2.55 g of the ANC-PE suspension without deionised water added. For all samples, a total mass of 5 g was prepared. Increasing the PE final concentration in samples was achieved by increasing the mass of ANC-PE suspension introduced into the hydrogel. However, the mass of deionised water added was reduced accordingly to respect the final mass of 5 g. Therefore, for all samples, final concentrations of 1.5% *w*/*w* CMC, 20% *w*/*w* glycerol and 1% *w*/*w* Cosgard^®^ were kept consistent. 

Overall, the mass of ANC-PE suspension added to hydrogel was between 0.121 g and 2.522 g. The amount of ultrapure water ranged from 2.409 g to 0 g. Ultimately, the 96 samples analysed had concentrations between 0.400% *w*/*w* PE and 8.369% *w*/*w* PE (Table 1). Considering the 0.1 mg accuracy of the analytical balance used to prepare the ANC-PE hydrogels, the errors of weighing were calculated to be ±0.08% for the lowest PE concentration and ±0.004% for the highest. Therefore, reference values used for the training of PLSR models were considered reliable and accurate.

### 2.2. Raman Spectroscopy Data Collection 

Raman analysis was performed with a LabRam spectrometer (Horiba Jobin-Yvon, Villeneuve-d’Ascq, France). A HeNe laser with a wavelength of 690 nm was used, and the power at the sample was close to ~10 mW. Backscattered light was collected with a ×10 microscope objective (Olympus, NA = 0.25). The confocal hole was set at 400 µm for all measurements. Detection was facilitated by dispersing Raman-shifted radiation onto a CCD detector using a grating (300 lines/mm), delivering a spectral resolution of ~4 cm^−1^ over the 150–3750 cm^−1^ spectral range. The acquisition time was 20 s. Considering the viscosity of the samples and the volumes available, it was convenient to place hydrogels into 96-well plates for data collection. The laser spot was manually focused below the surface of samples, from where the highest Raman intensities were collected. For each sample, 5 wells were filled and analysed. A total of 10 spectra were recorded for each well for a total of 50 spectra per sample. The final dataset had a total of 4800 Raman spectra from the 96 samples studied. Additionally, Raman spectra for each pure compound, i.e., sorbitan monooleate, polysorbate 80, sodium alginate, PE, glycerol and Cosgard^®^, were recorded with the same parameters.

### 2.3. Data Handling

**Figure 1:**
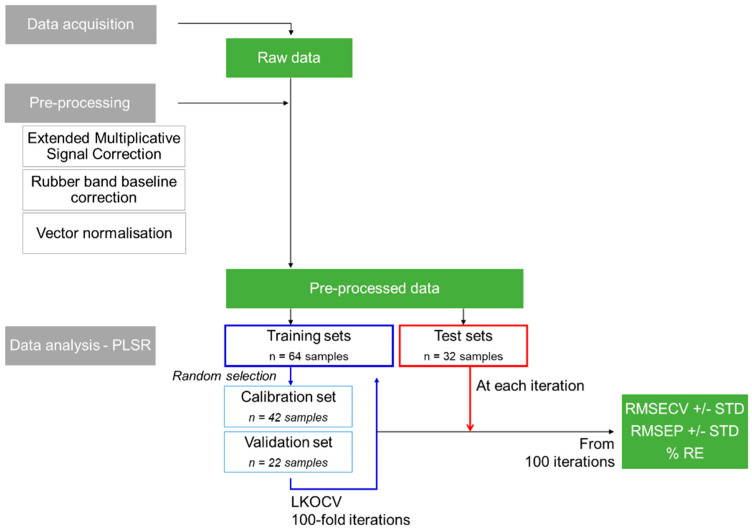
Overview of the data analysis protocol used.

The data mining was conducted with MATLAB^®^ (The Mathworks, Natick, MA, USA). The different steps are presented in Figure 1.

Firstly, Raman spectra were subjected to extended multiplicative signal correction (EMSC). The correction was applied using the freely available EMSC toolbox from Nofima Data Modelling (https://nofimadatamodeling.wordpress.com/software-downloads-list/) (accessed on 15 November 2021)). For additional details, see the tutorial published by Asfeth and Kohler [62]. In this study, a basic EMSC model was applied to the full spectral range of 150–3750 cm^−1^. Then, spectra were subjected to Rubber Band baseline correction followed by vector normalisation (RB-VN). Rubber Band is an algorithm computing a polynomial baseline to subtract from the data [63,64]. The method was applied with a polynomial order set to 1 to avoid over-correcting the spectra. The vector normalisation calculates the ratio of spectra to their respective Euclidian norms [29]. 

Principal component analysis (PCA) was used for data exploration. PCA is an unsupervised method used for data description and explorative data structure modelling [65,66]. PCA is widely used to simplify multidimensional datasets. It allows the reduction in the number of variables to retain the most important variation within the dataset [67,68]. The derivation of PC loadings, which represent the variance of each variable (wavenumber) for a given PC, gives information about variations in the chemical compositions, for instance, differences in spectra correlated to PE concentration [69]. 

Partial least squares regression (PLSR) was applied over the spectral range (300–3750 cm^−1^). PLSR correlates modifications in band positions, intensities and shapes with the systematically varying concentration of PE in hydrogel samples. PLSR is a supervised multivariate analysis method used for relating two sets of variables by quantifying them with respect to each other [65]. PLSR is extensively referenced in the literature as a robust and well-recognised quantitative approach used along with vibrational spectroscopy [65]. Therefore, PLSR is particularly powerful for constructing linear models from spectra collected from complex blends with many partially overlapping peaks. 

For the purpose of the study, each PLSR analysis was performed by splitting the dataset three ways. First, 2/3 of the dataset was used as the training set (=64 samples) and the remaining 1/3 of the dataset as the test set (=32 samples). Second, the training set was randomly sub-divided into the calibration set (*n* = 42 samples) and validation set (*n* = 22 samples) using a leave-K-out cross-validation (LKOCV) approach. SET 01, SET 02, SET 04, SET 05, SET 07, SET 08, SET 10 and SET 11 were constantly used as the training set. Purposely, the randomness of the LKOCV was kept in this study. Since there were multiple combinations of calibration/validation sets, a 100-fold iterative protocol was applied to challenge the dataset and provide an overall reliability estimate of the models (root mean squared error of cross-validation (RMSECV) and R^2^). Then, the test set (i.e., SET 03, SET 06, SET 09 and SET 12) was used in the predictive models as independent samples, i.e., previously unknown to the PLSR model, to be determined. At this stage, the performance of the predictive model has been assessed according to the root mean squared error of prediction (RMSEP), R^2^ and accuracy of the predicted concentration in percent relative error to the target (actual) concentration. In this study, predicted concentrations, RMSECV and RMSEP, are expressed as % *w*/*w* PE concentration.

## 3. Results and Discussion

### 3.1. Physicochemical Characterisation of ANC-PE Suspensions

ANC, and specifically ANC-PE, are spherical nanocarriers thoroughly described in previous publications [12,18,25]. In the present study, ANC-PE suspensions used to prepare the range of diluted samples are comparable to those previously obtained for the ATR-IR studies [25,26]. The average hydrodynamic diameter was 185 ± 8.5 nm, the polydispersity index was 0.109 ± 0.001 and the zeta potential was −25.8 ± 2.4 mV. These results show good reproducibility of the preparation protocol. The hydrodynamic diameter of the ANC-PE complies with the use of nano-systems in skin cosmetic products [70] as their size was between 100 and 200 nm. The polydispersity index of the nanocarriers is close to 0.1, which reflects a monodisperse suspension and the absence of aggregation. The zeta potential is close to −30 mV, which indicates good colloidal stability of the suspensions. ANC-PE-loaded hydrogels were white in colour, had a homogeneous aspect and did not show any sign of destabilisation over the course of the study. 

### 3.2. Characterisation of Spectral Variability According to ANC-PE Concentrations in the Dataset

The collected Raman signatures reflect the complex chemical nature of the samples analysed, consisting of combined contributions from the different ingredients used to prepare the hydrogel and the ANC-PE suspensions. 

**Figure 2:**
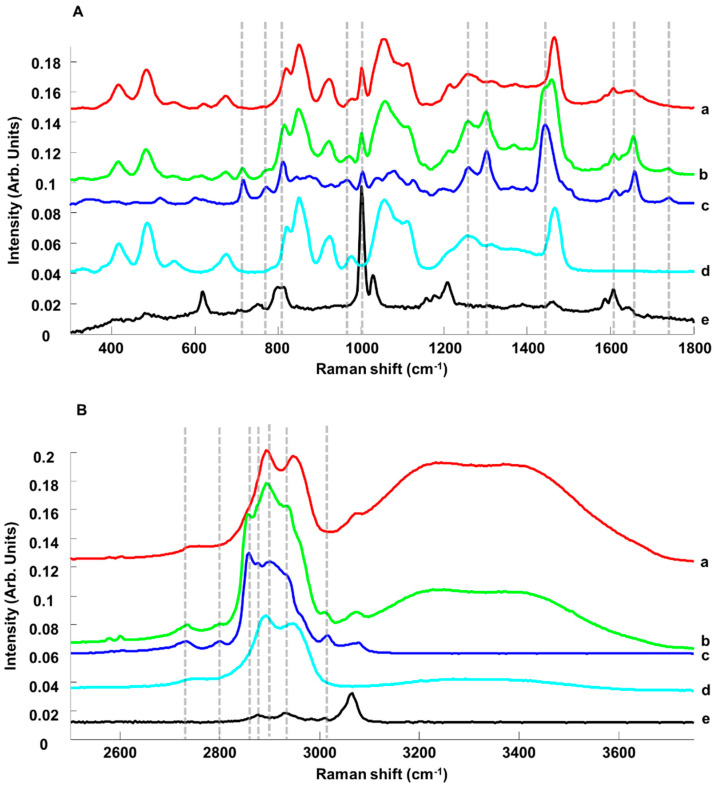
Mean Raman spectra in the range of 300–1800 cm^−1^ (**A**) and 2500–4000 cm^−1^ (**B**) collected from ANC-PE hydrogel SET 01-C1 (0.402% *w*/*w* PE) a, ANC-PE hydrogel SET 01-C8 (8.331% *w*/*w* PE) b, PE c, glycerol d and Cosgard^®^ e. Dotted lines indicate PE features. Spectra have been offset for clarity.

Figure 2 presents spectra collected from ANC-PE hydrogels SET 01-C1 (0.402% *w*/*w* PE) and SET 01-C8 (8.331% *w*/*w* PE), compared to mean spectra for pure ingredients, PE, glycerol and Cosgard^®^, in the fingerprint region (FPR) (Figure 2A) and high-wavenumber region (HWR) (Figure 2B). 

In the FPR (300–1800 cm^−1^), for PE (Figure 2A-c), the main features were observed at 715 cm^−1^ (=C-H bending), 771 cm^−1^ (=CH wagging), 812 cm^−1^ (=C-H bending), 844 cm^−1^, 877 cm^−1^, 925 cm^−1^ (=CH bending), 966 cm^−1^, 1004 cm^−1^, 1039 cm^−1^ (C-O, C-C stretching), 1080 cm^−1^ (C-C-C stretching), 1126 cm^−1^ (C-C-C stretching), 1258 cm^−1^ (C-O stretching), 1301 cm^−1^ (CH_2_ bending), 1442 cm^−1^ (CH_2_ deformation), 1612 cm^−1^ (C=O stretching), 1658 cm^−1^ (C=C stretching) and 1742 cm^−1^ (C=O stretching) [71]. For glycerol (Figure 2A-d), the main features were observed at 417 cm^−1^ (CCO rocking), 485 cm^−1^ (CCO rocking), 677 cm^−1^ (CCC deformation), 823 cm^−1^ (CC stretching), 850 cm^−1^ (CC stretching), 923 cm^−1^ (CH_2_ rocking), 977 cm^−1^ (CH_2_ rocking), 1055 cm^−1^ (CO stretching), 1109 cm^−1^ (CO stretching), 1258 cm^−1^ (C-O stretching) and 1466 cm^−1^ (CH_2_ deformation) [49,72]. For Cosgard^®^ (Figure 2A-e), the main features were found at 620 cm^−1^ (benzyl ring bending), 801 cm^−1^ (C-C stretching), 1001 cm^−1^ (benzyl ring stretching), 1028 cm^−1^ (in-plane CH bending), 1207 cm^−1^ (C-C stretching), 1588 cm^−1^ (C-O stretching) and 1609 cm^−1^ (quadrant benzyl ring stretching) [71].

In the HWR (2500–3750 cm^−1^), PE features were observed at 2731 cm^−1^ (CH stretching), 2801 cm^−1^ (CH stretching), 2858 cm^−1^ (symmetric CH_2_ stretching), 2901 cm^−1^ (antisymmetric CH_2_ stretching), 3015 cm^−1^ (=CH stretching) and 3080 cm^−1^ (=CH stretching) (Figure 2B-c) [71]. For glycerol, bands at 2742 cm^−1^ (CH stretching from C2), 2893 cm^−1^ (symmetric CH_2_ stretching) and 2947 cm^−1^ (antisymmetric CH_2_ stretching), and a broad band with a maximum of 3326 cm^−1^ (symmetric and antisymmetric OH stretching) was observed (Figure 2B-d) [49,72]. For Cosgard^®^, weak features at 2877 cm^−1^ (CH stretching), 2928 cm^−1^ (CH stretching) and 3066 cm^−1^ (= CH stretching) were found (Figure 2B-e) [71]. 

The comparison of mean normalised spectra from ANC-PE hydrogel samples SET 01-C1 (0.402% *w*/*w* PE) and SET 01-C8 (8.331% *w*/*w* PE) highlighted a number of spectral variations. While for the sample of 0.402% *w*/*w* PE, the contributions from glycerol (Figure 2A-d) and Cosgard^®^ (Figure 2A-e) are quite noticeable, for the sample of 8.331% *w*/*w* PE, the main features from PE exhibited higher intensities (Figure 2A-c dotted lines). In the HWR, it was also observed that the spectral signature for the concentration 8.331% *w*/*w* PE (Figure 2B-b) displayed a significant contribution from PE (Figure 2B-c) compared to the hydrogel at 0.402% *w*/*w* PE (Figure 2B-a). Moreover, the water features at 3080–3750 cm^−1^ (symmetric and antisymmetric OH stretching) are mostly observed for low PE concentrations, and the contribution decreases in intensity as the ACI content increases.

### 3.3. Principal Component Analysis (PCA)

**Figure 3:**
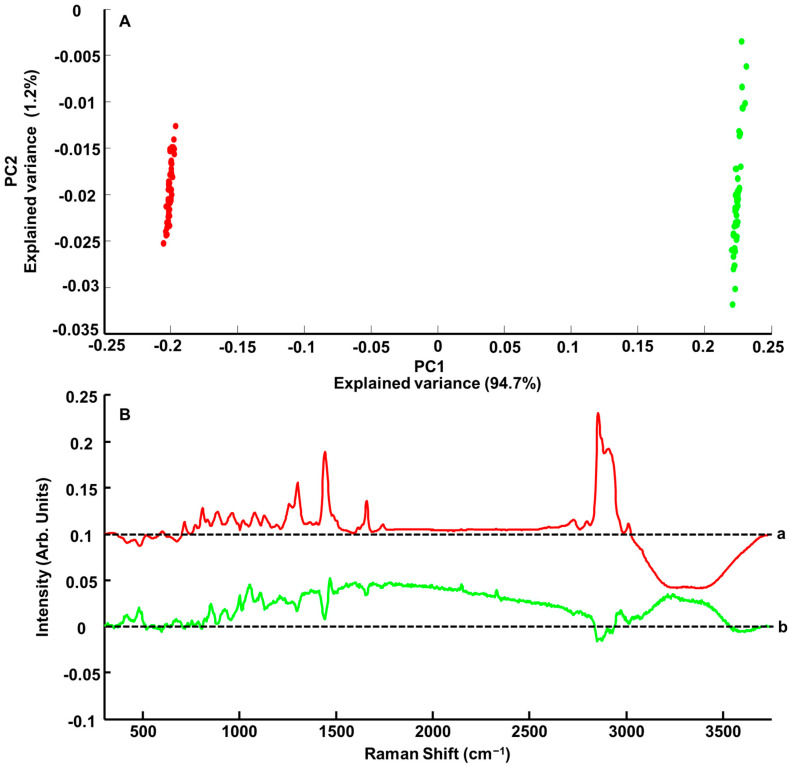
(**A**) Scatter plot (PC1 versus PC2) from PCA analysis performed on pre-processed Raman spectra collected from ANC-PE hydrogel SET 01-C1 (0.402% *w*/*w* PE) (red dots) and ANC-PE hydrogel SET 01-C8 (8.331% *w*/*w* PE) (green dots). (**B**) PC loading 1 a, and PC loading 2 b over the range of 300–3750 cm^−1^. Dotted lines indicate the zero level. Spectra have been offset for clarity.

PCA is a relevant approach for data visualisation, allowing the identification of spectral features with the greatest variability in the collected datasets. Figure 3A displays an example of the PCA scatterplot obtained from SET-01 samples with the highest and lowest PE concentrations, respectively, of 0.402% *w*/*w* PE (Figure 3A red dots) and 8.331% *w*/*w* PE (Figure 3A green dots). The two sets of spectra are well separated based on PC1, which accounts for 94.7% of the explained variance, while PC2 accounts for only 1.2%.

The loadings of the principal components are shown in Figure 3B. In the FPR, PC1 (Figure 3B-a) has positive peaks at 715 cm^−1^, 774 cm^−1^, 809 cm^−1^, 887 cm^−1^, 963 cm^−^, 1077 cm^−1^, 1128 cm^−1^, 1258 cm^−1^, 1301 cm^−1^, 1442 cm^−1^, 1658 cm^−1^ and 1739 cm^−1^ that can be matched to features in the pure spectrum of PE (Figure 2A-c). The negative bands at 417 cm^−1^, 482, and 677 cm^−1^ are assigned to glycerol (Figure 2A-d). In the HW region, the positive peaks in PC1 at 2726 cm^−1^, 2799 cm^−1^, 2855 cm^−1^, 2909 cm^−1^ and 3012 cm^−1^ are assigned to PE (Figure 2A-c). The broad negative band in the range of 3080–3750 cm^−1^ is a characteristic feature from OH vibrations that can be, in the present case, mainly attributed to the water added to samples (see Section 2). 

Along PC2 (Figure 3A), it is observed that data from both concentrations are scattered. The green dots corresponding to the highest concentration are more spread out, but no discrimination can be observed in that direction. Although in the FPR of the PC2 loading (Figure 3B-b), positive features observed at 417 cm^−1^, 479 cm^−1^, 852 cm^−1^, 923 cm^−1^, 1053 cm^−1^, 1109 cm^−1^ and 1469 cm^−1^ can be assigned to glycerol (Figure 2A-d) and positive bands at 1001 cm^−1^ and 1212 cm^−1^ to Cosgard^®^ (Figure 2A-e), the shape of the baseline strongly suggests the presence of residual background in the data (see dotted line representing the zero line). The HWR is also affected, but the broad positive feature is also attributed to modification in the water region, while negative features at 2855 cm^−1^ and 2915 cm^−1^ can be residual PE contributions (Figure 2A-c). The loading of PC2 highlights the heterogeneity in the data collected, although the explained variance of 1.2% suggests that it is weak compared to the increase in spectral features intensities related to systematically varying concentrations of PE. 

### 3.4. Construction of Predictive Models (Cross-Validation)

As described in Section 2.3, data were split three ways into the calibration set (*n* = 42 samples), the validation set (*n* = 22 samples) and the test set (*n* = 32 samples).

**Figure 4:**
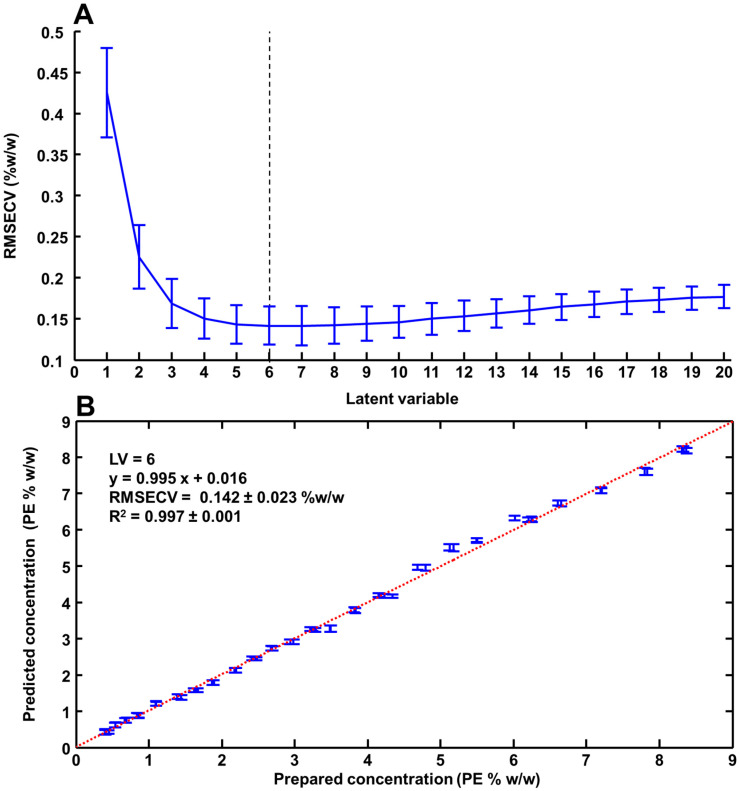
(**A**) RMSECV as a function of number of latent variables. (**B**) Prepared PE concentrations regressed against predicted PE concentration from the training set (cross-validation). Error bars represent the mean ± standard deviation for each validation sample.

Figure 4A presents the RMSECV as a function of the number of latent variables (LVs) obtained from 100 iterations of cross-validation. The mean RMSECV displayed a decrease for the first 6 LVs to reach a minimum value at 0.142 ± 0.023% *w*/*w* PE, followed by an increase up to 0.177 ± 0.014% *w*/*w* PE at (LV = 20). For this study, LV = 6 has been selected, considering that beyond this point, no improvement in the reliability of the quantification could be achieved.

Figure 4B shows a plot of prepared % *w*/*w* PE concentration regressed against % *w*/*w* PE concentrations predicted from 100 iterations of cross-validation. The R^2^ = 0.997 ± 0.001 indicates a good correlation, despite the fact that samples of the highest concentrations appear to be more scattered around the linear regression line. The RMSECV of 0.142% *w*/*w* PE corresponds to ~3.6% of the median concentration (3.985% *w*/*w* PE) of the range studied.

**Figure 5:**
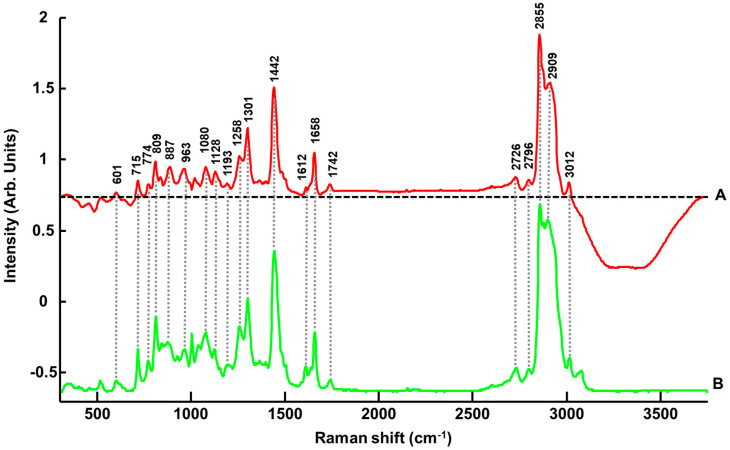
First regression coefficient from PLSR analysis performed on Raman spectra collected from ANC-PE hydrogels (A) compared to the reference spectrum for PE (B). Dotted line indicates the zero baseline.

The regression coefficient obtained from PLSR analysis performed on Raman spectra collected from ANC-PE hydrogels (Figure 5A) compared to the spectrum of PE (Figure 5B) confirms the high molecular specificity of the predictive model to detect and quantify spectral variations of the ACI. Positive features in the FPR at 601 cm^−1^, 715 cm^−1^, 774 cm^−1^, 809 cm^−1^, 887 cm^−1^, 963 cm^−1^, 1080 cm^−1^, 1128 cm^−1^, 1193 cm^−1^, 1258 cm^−1^, 1301 cm^−1^, 1442 cm^−1^, 1612 cm^−1^, 1658 cm^−1^ and 1742 cm^−1^ correspond to the main PE features (Figure 5B), already seen in the PC1 loading from the PCA (Figure 3B-a). Negative features (Figure 5A) at 417 cm^−1^, 482 cm^−1^ and 677 cm^−1^ are assigned to glycerol (Figure 2A-d). In the HWR, the same observation was made, with positive bands at 2726 cm^−1^, 2796 cm^−1^, 2855 cm^−1^, 2909 cm^−1^ and 3012 cm^−1^ corresponding to PE, while the broad water bands in the range of 3080–3750 cm^−1^ are negative. The water concentration is inversely proportional to the ANC-PE concentration in hydrogels, and, therefore, the predictive model relies on the variations in band ratios to create a correlation with the absolute concentration of ACI in samples. The same explanation applies to glycerol that is found in hydrogel samples at a constant concentration of 20% *w*/*w* (see Section 2.1.3), hence the anti-correlated evolution of its features compared to PE.

Table 2 gives a summary of the percent relative error for the 64 samples used as the training set. The percent relative error (%RE) ranged from 0.14% to 12.07% and the mean % relative error is 3.58%. PE concentrations (% *w*/*w*) were determined with a relative error inferior to 5% for 46 out of 64 samples analysed, and 16 samples were predicted with a relative error between 5 and 10%. Two samples were determined with % RE higher than 10%, i.e., 11.15% for SET-04-C1 (0.537% *w*/*w* PE) and 12.07% for SET-10-C1 (0.543% *w*/*w* PE).

Table 3 provides another representation of the distribution of % RE according to PE concentrations. The percentage relative errors higher than 7.5%, i.e., 12.07% for SET-10-C1 (0.543% *w*/*w* PE), 11.15% for SET-04-C1 (0.537% *w*/*w* PE), 9.86% for SET-11-C1 (0.668% *w*/*w* PE), 9.86% for SET-10-C2 (1.096% *w*/*w* PE), 9.64% for SET-02-C1 (0.452% *w*/*w* PE), 7.94% for SET-04-C2 (1.102% *w*/*w* PE) and 7.50% for SET-05-C1 (0.694% *w*/*w* PE), indicate that the highest errors were obtained for samples with the lowest PE concentrations (i.e., C1 and C2 from different sets). For samples above 2% *w*/*w* PE, the relative error remained below 5%, except for samples SET 11-C6 (5.123% *w*/*w*) (%RE = 7.31%), SET-04-C5 (3.494% *w*/*w*) (%RE = 6.43%), SET-10-C5 (3.483% *w*/*w*) (%RE = 6.16%), SET-05-C6 (5.180% *w*/*w*) (%RE = 6.01%) and SET-10-C6 (4.684% *w*/*w*) (%RE = 5.73%). It is, however, observed that %RE remains ~5–7% for these samples. The overall mean %RE = 3.58% suggests that Raman spectroscopy has the ability to accurately predict PE concentration in hydrogel samples.

### 3.5. Prediction of PE Concentration in Test Samples (Unknown; to Be Determined)

**Figure 6:**
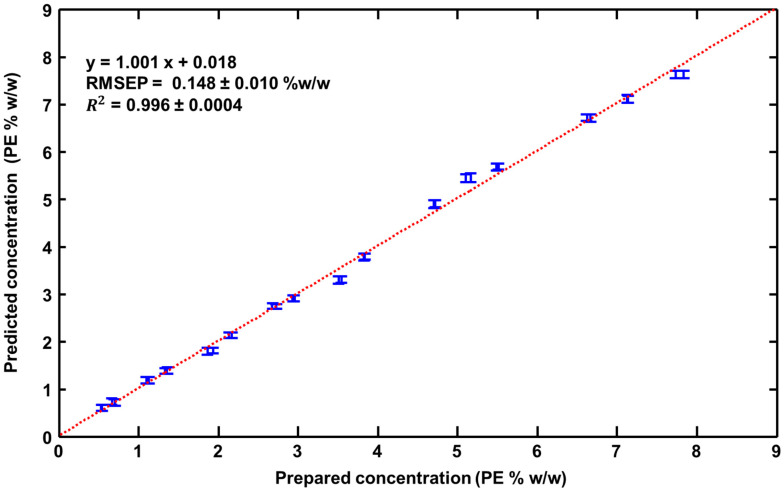
Prepared PE concentrations regressed against predicted PE concentrations for the test set (Unknown; to be determined). Error bars represent the mean ± standard deviation for each test sample.

Figure 6 presents the regression plot obtained from the PLSR analysis performed on the test samples (SET 03, SET 06, SET 09 and SET 12) that were projected into the predictive model as unknown samples to be determined. The results were RMSEP = 0.148 ± 0.010% *w*/*w* PE and R^2^ = 0.996 ± 0.0004 (with LV = 6). The RMSEP corresponds to ~3.7% of the median concentration (3.985% *w*/*w*) of the range studied.

Table 4 gives a summary of %RE for the 32 test samples analysed. The values range from 0.22% to 11.58%, and the mean % relative error is 3.67%. PE concentrations (% *w*/*w*) were determined with a relative error inferior to 5% for 23 out of 32 samples analysed. %RE from 5 to 7.5% were obtained with 6 samples. Three samples have a % relative error above 10%: SET-03-C1 (0.533% *w*/*w* PE) (11.58%), SET-09-C1 (0.542% *w*/*w* PE) (10.29%) and SET-06-C1 (0.667% *w*/*w* PE) (10.09%). Similarly, to the training set, the highest %RE is found in samples with the lowest PE concentrations (C1–C2) (Table 5).

### 3.6. General Discussion

Similar to ATR-IR spectroscopy, confocal Raman microspectroscopy provides a specific molecular fingerprint of the cosmetic hydrogels under investigation. The spectra result from the combined contribution of ingredients in the formula, correlated to their respective concentrations. For both techniques, the regression coefficients obtained from PLSR analysis highlighted the contributions of specific features from PE to the predictive models, as well as the presence of other bands from ingredients such as glycerol and water (Figure 5 and [25,26] for ATR-IR spectra). The anti-correlated patterns between the increasing intensities of PE spectral bands according to their final concentration in hydrogels and the decreasing intensities of water and glycerol bands were found to be identical for both methods.

A direct comparison of Raman analysis with results previously obtained using ATR-IR spectroscopy [26] is not possible due to the differing number of samples, concentration range and spectral range used to apply the PLSR analysis. Nevertheless, the physicochemical characteristics of ANC-PE suspensions (average hydrodynamic diameter, polydispersity index and zeta potential) for ANC-PE suspensions used to prepare the range of diluted samples were found to be very similar to values previously obtained for the ATR-IR study [25,26]. Furthermore, the loaded ANC-PE hydrogels were prepared with standardised experimental conditions (same instruments, protocols and ingredients); therefore, for both studies, they are highly comparable. The respective mean %RE of 2.51% and 3.67% calculated for the test sets for ATR-IR and for Raman, respectively, suggest a similar level of accuracy in the prediction of unknown samples. Although slightly different values were obtained for RMSECV = 0.097% *w*/*w* PE (R^2^ = 0.995) for ATR-IR compared to RMSECV = 0.142% *w*/*w* PE (R^2^ = 0.997) for Raman (Figure 4), both techniques can be considered suitable for rapid and cost-effective analytical control of complex cosmetic formulations. Beyond their respective performance to quantify PE in cosmetic hydrogels, the applicability and/or transferability to either academic research laboratories or the industrial environment should be considered. On the one hand, ATR-IR spectroscopy is a highly robust and reliable (alignment, calibration and requiring minimal maintenance) technique that has been employed to analyse liquid samples such as human body fluids [73,74], chemotherapeutic drugs [51,52,61,75] and natural deep eutectic solvents (NADES) [76,77], either as liquids or after drying. The upgrade from measurements performed in transmission mode to the ATR mode, thanks to the availability of compact accessories, has enabled rapid and easy analysis from a single drop of the sample deposited onto a flat ATR crystal (commonly diamond) [78]. ATR-IR setups most commonly found in analytical labs remain, however, most suited for analysis of reduced batches of samples, considering the workflow that requires cleaning the ATR crystal and recording a new background between each measurement. High-throughput ATR plate readers (e.g., Pike Technologies) [79,80] open up the possibility to prepare samples remotely and run automated batch analysis, although the use of such systems is not yet widespread. Quantum cascade laser IR spectroscopy is an emerging alternative, using brighter light sources for increased analysis speed and sensitivity [81]. Despite the high cost of such an apparatus, these innovations contribute to strengthening the case for applications of infrared spectroscopy for analytical quality control (ACQ).

In comparison, Raman spectroscopy is still considered a complex technique for expert users. However, measurements through quartz cuvettes can easily be performed with a macro setup, highlighting the feasibility of performing quantitative analysis through a container. Makki et al. have reported the AQC of chemotherapeutic solutions with systematically varying concentrations of drugs in quartz cuvettes [52], while Elderderi et al. have conducted similar studies of NADES to quantify water content [48]. In the last few years, considerable efforts to miniaturise systems have resulted in more affordable transportable (benchtop) devices with remote probes and compact handheld devices. Most commercial Raman instruments are designed for operation in confocal microscopic conditions. In such setups, the technique measures backscattered light, and therefore, the laser source does not have to go through the sample. The technique of spatially offset Raman spectroscopy (SORS) uses a central fibre to deliver the source laser, and spatially offset fibres to collect the backscattered Raman signal, exploiting the fact that the offset signal originates from deeper in the sample, enabling the measurement of larger volumes (e.g., larger packaging containers [82,83,84], blister packs [85], vials [49,59] or other containers such as perfusion bags for chemotherapeutic drugs [60,86]). SORS is a more user-friendly approach, which does not require a high degree of user training. Nevertheless, a thorough evaluation of the application as a quantitative analytical tool is necessary to validate its suitability to screen complex cosmetic products. The main benefits of in situ analysis are to guarantee sample integrity while avoiding contamination and to significantly improve workflow while offering a 100% consumable-free alternative. Another benefit of Raman spectroscopy is that, without the requirements for consumables, it can perform in-line quantitative ACQ, either using immersed fibre probes [87,88] or through a (transparent) vessel wall [87,89].

In terms of application potential, the PLSR outcome for the Raman analysis could be significantly improved through enhanced repeatability and reproducibility of the measurement. In this preliminary study, the laser was manually focused just below the surface of the samples. In terms of repeatability, error bars in Figure 4 correspond to the standard deviation calculated from the 50 predicted PE concentration obtained from the 50 spectra recorded per sample of the validation set (see Section 2). The mean standard deviation was found to be 0.08 +/− 0.021% *w*/*w* PE. The coefficient of variations expressed in percent (CV%) were ranging between 1.10% and 18.4% with a mean value equal to 4.14%. It was observed that the highest CV% was found for samples with concentrations below 1% *w*/*w* PE, suggesting that the repeatability in the measurements is lower most likely due to weaker intensities, and hence a lower signal-to-noise ratio, from the AI compared to other ingredients. Above 1% *w*/*w* PE, the CV% decreased rapidly, and it was found inferior to 2% for the highest concentration, i.e., sample with more than% *w*/*w* PE. Similar observations were made for the test set (Figure 6) with a mean standard deviation of 0.077 ± 0.010% *w*/*w* PE and CV% ranging from 1.07% to 11.8% (mean = 3.67%). Automated sample positioning and light collection could help to standardise the data collection process and to maximise the signal-to-noise ratio at every single analysis. Commercially available confocal Raman microscopes typically include an autofocus feature, so it is easy to develop an automated routine to automatically set the zero position and then increment a predefined motion to focus the laser to a constant depth within the sample. Raman microscopes can be purchased with an inverted configuration (objective lens pointing to the ceiling) that can be used to analyse liquid samples [90,91], and the technique lacks a dedicated instrumental setup for liquid diffracting samples. Moreover, a number of parameters such as the wavelength of the laser source and magnification of the focusing objectives must be considered and compared to determine if Raman spectroscopy is suitable for quality control of cosmetic hydrogels.

In the context of determining ACI concentration in complex cosmetic forms, the direct assay of samples without the need for pre-analytical steps and the absence of solvent or consumable requirements are indisputable advantages over chromatography-based protocols. Spectroscopic analysis offers the advantages of reagentless and thus more eco-friendly techniques that can be implemented in the field in an industrial environment. Indeed, a more thorough evaluation of the technique should be performed according to established guidelines. For instance, the ICH Q2(R2) validation of analytical procedures from the European Medicines Agency provides additional criteria such as intermediate precision or inter-laboratory reproducibility that should be determined from larger datasets [92]. Ultimately, Raman spectroscopy is a versatile technique that can be adapted to perform analysis outside, through or inside containers, which is a significant advantage over ATR-IR. It is indeed necessary to screen larger panels of systems with various chemical compositions to have a comprehensive view of the advantages and limitations of both vibrational techniques evaluated.

## 4. Conclusions

Raman spectroscopy is a powerful technique for label-free molecular characterisation of samples. The molecular specificity allows us to detect and quantify the ACI in complex semi-solid formulations with no requirements for pre-analytical protocols. Although PE is nanoencapsulated in core–shell nanocarriers, Raman analysis coupled with data mining protocols such as PLSR resulted in robust quantitative models with an RMSEP of 0.148% *w*/*w* and R^2^ of 0.996. With a mean % error equal to 3.67% for the 32 predicted-as-unknown samples, the feasibility to deliver accurate quantification is highlighted. While optimisation in the experimental setup and sample presentation for higher reproducibility need to be sought, the results obtained in the present study showed the potential of Raman spectroscopy as a promising tool for the analytical quality control of cosmetic products.

## Figures and Tables

**Table 1:** Final PE concentration (% *w*/*w*) in samples analysed.

PE Final Concentration (% *w*/*w*)								
**Concentration**	C1	C2	C3	C4	C5	C6	C7	C8
**SET 01**	0.402	0.857	1.671	2.481	3.285	4.231	6.255	8.331
**SET 02**	0.452	0.837	1.640	2.411	3.213	4.341	6.016	8.306
**SET 03**	0.533	1.128	1.865	2.727	3.507	4.703	5.513	7.127
**SET 04**	0.537	1.102	1.870	2.679	3.494	4.794	5.493	7.194
**SET 05**	0.694	1.391	2.197	2.986	3.833	5.180	6.657	7.833
**SET 06**	0.667	1.333	2.167	2.933	3.833	5.167	6.667	7.833
**SET 07**	0.400	0.835	1.617	2.461	3.276	4.149	6.249	8.331
**SET 08**	0.408	0.861	1.667	2.459	3.244	4.158	6.203	8.369
**SET 09**	0.542	1.103	1.934	2.678	3.539	4.720	5.486	7.134
**SET 10**	0.543	1.097	1.890	2.696	3.483	4.684	5.497	7.209
**SET 11**	0.668	1.443	2.177	2.938	3.816	5.123	6.607	7.799
**SET 12**	0.702	1.360	2.134	2.954	3.826	5.102	6.620	7.737

**Table 2:** Summary of percent relative errors for the training set (*n* = 64).

%RE	<1	<2.5	<5	<7.5	<10	<12.5
**Number of samples**	9	22	15	11	5	2
**Min (%RE)**	0.14	1.02	2.54	5.41	7.59	11.15
**Max (% RE)**	0.95	2.49	4.99	7.31	9.86	12.07
**Mean (%RE)**	0.61	1.54	3.53.	6.25	8.98	11.61
**Overall mean (%RE)**	3.58					

**Table 3:** Summary of mean % relative errors according to PE concentration ranges (training set).

PE Concentration (*w*/*w*%)	No of Samples	Mean %RE	Min–Max %RE
<1	12	6.57	1.22–12.07
[1, 2]	10	5.21	0.55–9.86
[2, 3]	10	1.77	0.91–3.74
[3, 4]	8	2.44	0.95–6.43
[4, 5]	6	2.63	0.49–5.73
[5, 6]	4	5.13	3.46–7.31
[6, 7]	6	1.52	0.19–4.76
[7, 8]	4	2.35	1.54–3.14
>8	4	1.52	1.02–2.28

**Table 4:** Summary of percent relative errors for the test set.

%RE	<1	<2.5	<5	<7.5	<10	<12.5
**Number of samples**	6	8	9	6	0	3
**Min (%RE)**	0.22	1.19	2.72	5.48	-	10.09
**Max (%RE)**	0.69	2.23	4.77	6.82	-	11.58
**Mean (%RE)**	0.39	1.61	3.57	6.34	-	10.65
**overall mean (%RE)**	3.67					

**Table 5:** Summary of mean % relative errors according to PE concentration ranges (test set).

PE Concentration	No. of Samples	Mean RE%	Min–Max %RE
<1	4	8.76	3.07–11.57
[1, 2]	6	4.61	2.09–6.82
[2, 3]	6	1.12	0.33–2.24
[3, 4]	4	3.75	1.19–6.67
[4, 5]	2	3.89	3.75–4.03
[5, 6]	4	4.64	3.02–6.64
[6, 7]	2	0.977	0.59–1.36
[7, 8]	4	1.17	0.18–2.72
>8	-	-	-

## Data Availability

The data presented in this study are available on request from the corresponding author.

## References

[1] Jackson L.M., Schwinn D.A., National Academies of Sciences, Engineering, and Medicine, Health and Medicine Division, Board on Health Sciences Policy, Committee on the Assessment of the Available Scientific Data Regarding the Safety and Effectiveness of Ingredients Used in Compounded Topical Pain Creams (2020). Commissioned Paper: Topical Dosage Form Development and Evaluation.

[2] Garg T., Rath G., Goyal A.K. (2015). Comprehensive Review on Additives of Topical Dosage Forms for Drug Delivery. Drug Deliv..

[3] Peppas N.A., Bures P., Leobandung W., Ichikawa H. (2000). Hydrogels in Pharmaceutical Formulations. Eur. J. Pharm. Biopharm..

[4] Bielfeldt S., Bonnier F., Byrne H.J., Chourpa I., Dancik Y., Lane M.E., Lunter D.J., Munnier E., Puppels G., Tfayli A. (2022). Monitoring Dermal Penetration and Permeation Kinetics of Topical Products; the Role of Raman Microspectroscopy. TrAC Trends Anal. Chem..

[5] Nafisi S., Maibach H.I., Sakamoto K., Lochhead R.Y., Maibach H.I., Yamashita Y. (2017). Nanotechnology in Cosmetics. Cosmetic Science and Technology.

[6] Zhou H., Luo D., Chen D., Tan X., Bai X., Liu Z., Yang X., Liu W. (2021). Current Advances of Nanocarrier Technology-Based Active Cosmetic Ingredients for Beauty Applications. Clin. Cosmet. Investig. Dermatol..

[7] Elmowafy M. (2021). Skin Penetration/Permeation Success Determinants of Nanocarriers: Pursuit of a Perfect Formulation. Colloids Surf. B Biointerfaces.

[8] Hazari S.A., Kaur H., Karwasra R., Abourehab M.A., Khan A.A., Kesharwani P. (2023). An Overview of Topical Lipid-Based and Polymer-Based Nanocarriers for Treatment of Psoriasis. Int. J. Pharm..

[9] Luo W., Bai L., Zhang J., Li Z., Liu Y., Tang X., Xia P., Xu M., Shi A., Liu X. (2023). Polysaccharides-Based Nanocarriers Enhance the Anti-Inflammatory Effect of Curcumin. Carbohydr. Polym..

[10] Yao L., Xu J., Zhang L., Liu L., Zhang L. (2021). Nanoencapsulation of Anthocyanin by an Amphiphilic Peptide for Stability Enhancement. Food Hydrocoll..

[11] Yi J., He Q., Peng G., Fan Y. (2022). Improved Water Solubility, Chemical Stability, Antioxidant and Anticancer Activity of Resveratrol via Nanoencapsulation with Pea Protein Nanofibrils. Food Chem..

[12] Nguyen H.T.P., Munnier E., Souce M., Perse X., David S., Bonnier F., Vial F., Yvergnaux F., Perrier T., Cohen-Jonathan S. (2015). Novel Alginate-Based Nanocarriers as a Strategy to Include High Concentrations of Hydrophobic Compounds in Hydrogels for Topical Application. Nanotechnology.

[13] Park S.J., Garcia C.V., Shin G.H., Kim J.T. (2017). Development of Nanostructured Lipid Carriers for the Encapsulation and Controlled Release of Vitamin D3. Food Chem..

[14] Oliveira C., Coelho C., Teixeira J.A., Ferreira-Santos P., Botelho C.M. (2022). Nanocarriers as Active Ingredients Enhancers in the Cosmetic Industry—The European and North America Regulation Challenges. Molecules.

[15] Müller R.H., Radtke M., Wissing S.A. (2002). Solid Lipid Nanoparticles (SLN) and Nanostructured Lipid Carriers (NLC) in Cosmetic and Dermatological Preparations. Adv. Drug Deliv. Rev..

[16] Roberts M., Mohammed Y., Pastore M., Namjoshi S., Yousef S., Alinaghi A., Haridass I., Abd E., Leite-Silva V., Benson H. (2017). Topical and Cutaneous Delivery Using Nanosystems. J. Control. Release.

[17] Nguyen H.T.P., Soucé M., Perse X., Vial F., Perrier T., Yvergnaux F., Chourpa I., Munnier E. (2017). Lipid-Based Submicron Capsules as a Strategy to Include High Concentrations of a Hydrophobic Lightening Agent in a Hydrogel. Int. J. Cosmet. Sci..

[18] Nguyen H.T.P., Allard-Vannier E., Gaillard C., Eddaoudi I., Miloudi L., Soucé M., Chourpa I., Munnier E. (2016). On the Interaction of Alginate-Based Core-Shell Nanocarriers with Keratinocytes in Vitro. Colloids Surf. B Biointerfaces.

[19] Boutin R., Munnier E., Renaudeau N., Girardot M., Pinault M., Chevalier S., Chourpa I., Clément-Larosière B., Imbert C., Boudesocque-Delaye L. (2019). Spirulina Platensis Sustainable Lipid Extracts in Alginate-Based Nanocarriers: An Algal Approach against Biofilms. Algal Res..

[20] Managuli R.S., Kumar L., Chonkar A.D., Shirodkar R.K., Lewis S., Koteshwara K.B., Reddy M.S., Mutalik S. (2016). Development and Validation of a Stability-Indicating RP-HPLC Method by a Statistical Optimization Process for the Quantification of Asenapine Maleate in Lipidic Nanoformulations. J. Chromatogr. Sci..

[21] Temova Rakuša Ž., Škufca P., Kristl A., Roškar R. (2021). Quality Control of Retinoids in Commercial Cosmetic Products. J. Cosmet. Dermatol..

[22] Gałuszka A., Migaszewski Z., Namieśnik J. (2013). The 12 Principles of Green Analytical Chemistry and the SIGNIFICANCE Mnemonic of Green Analytical Practices. TrAC Trends Anal. Chem..

[23] Kommalapati H.S., Pilli P., Samanthula G. (2023). Green Sample Preparation in Bioanalysis: Where Are We Now?. Bioanalysis.

[24] Prajapati P., Rajpurohit P., Pulusu V.S., Shah S. (2023). Green and Sustainable Analytical Chemistry-Driven Chromatographic Method Development for Stability Study of Apixaban Using Box–Behnken Design and Principal Component Analysis. J. Chromatogr. Sci..

[25] Miloudi L., Bonnier F., Barreau K., Bertrand D., Perse X., Yvergnaux F., Byrne H.J., Chourpa I., Munnier E. (2018). ATR-IR Coupled to Partial Least Squares Regression (PLSR) for Monitoring an Encapsulated Active Molecule in Complex Semi-Solid Formulations. Analyst.

[26] Bonnier F., Miloudi L., Henry S., Bertrand D., Tauber C., Perse X., Yvergnaux F., Byrne H.J., Chourpa I., Munnier E. (2020). Quantification of Low-Content Encapsulated Active Cosmetic Ingredients in Complex Semi-Solid Formulations by Means of Attenuated Total Reflectance-Infrared Spectroscopy. Anal. Bioanal. Chem..

[27] Byrne H., Sockalingum G., Stone N. (2011). Raman Microscopy: Complement or Competitor?. RSC Anal. Spectrosc. Ser..

[28] Pirutin S.K., Jia S., Yusipovich A.I., Shank M.A., Parshina E.Y., Rubin A.B. (2023). Vibrational Spectroscopy as a Tool for Bioanalytical and Biomonitoring Studies. Int. J. Mol. Sci..

[29] Butler H.J., Ashton L., Bird B., Cinque G., Curtis K., Dorney J., Esmonde-White K., Fullwood N.J., Gardner B., Martin-Hirsch P.L. (2016). Using Raman Spectroscopy to Characterize Biological Materials. Nat. Protoc..

[30] Wang Y., Peng H., Liu K., Shang L., Xu L., Lu Z., Li B. (2023). Multi-Point Scanning Confocal Raman Spectroscopy for Accurate Identification of Microorganisms at the Single-Cell Level. Talanta.

[31] Uematsu M., Shimizu T. (2021). Raman Microscopy-Based Quantification of the Physical Properties of Intracellular Lipids. Commun. Biol..

[32] Auner G.W., Koya S.K., Huang C., Broadbent B., Trexler M., Auner Z., Elias A., Mehne K.C., Brusatori M.A. (2018). Applications of Raman Spectroscopy in Cancer Diagnosis. Cancer Metastasis Rev..

[33] Vlasov A.V., Maliar N.L., Bazhenov S.V., Nikelshparg E.I., Brazhe N.A., Vlasova A.D., Osipov S.D., Sudarev V.V., Ryzhykau Y.L., Bogorodskiy A.O. (2020). Raman Scattering: From Structural Biology to Medical Applications. Crystals.

[34] Stella A., Bonnier F., Tfayli A., Yvergnaux F., Byrne H.J., Chourpa I., Munnier E., Tauber C. (2020). Raman Mapping Coupled to Self-Modelling MCR-ALS Analysis to Estimate Active Cosmetic Ingredient Penetration Profile in Skin. J. Biophotonics.

[35] Wang H., Li J., Qin J., Li J., Chen Y., Song D., Zeng H., Wang S. (2021). Confocal Raman Microspectral Analysis and Imaging of the Drug Response of Osteosarcoma to Cisplatin. Anal. Methods.

[36] Depciuch J., Kaznowska E., Zawlik I., Wojnarowska R., Cholewa M., Heraud P., Cebulski J. (2016). Application of Raman Spectroscopy and Infrared Spectroscopy in the Identification of Breast Cancer. Appl. Spectrosc..

[37] Ren J., Mao S., Lin J., Xu Y., Zhu Q., Xu N. (2022). Research Progress of Raman Spectroscopy and Raman Imaging in PharmaceuticalAnalysis. Curr. Pharm. Des..

[38] Shaikh R., Daniel A., Lyng F.M. (2023). Raman Spectroscopy for Early Detection of Cervical Cancer, a Global Women’s Health Issue—A Review. Molecules.

[39] Mohamed H.T., Untereiner V., Proult I., Ibrahim S.A., Götte M., El-Shinawi M., Mohamed M.M., Sockalingum G.D., Brézillon S. (2018). Characterization of Inflammatory Breast Cancer: A Vibrational Microspectroscopy and Imaging Approach at the Cellular and Tissue Level. Analyst.

[40] Rammal H., Al Assaad A., Dosio F., Stella B., Maksimenko A., Mura S., Van Gulick L., Callewaert M., Desmaële D., Couvreur P. (2021). Investigation of Squalene-Doxorubicin Distribution and Interactions within Single Cancer Cell Using Raman Microspectroscopy. Nanomed. Nanotechnol. Biol. Med..

[41] Byrne H.J., Bonnier F., Casey A., Maher M., McIntyre J., Efeoglu E., Farhane Z. (2018). Advancing Raman Microspectroscopy for Cellular and Subcellular Analysis: Towards in Vitro High-Content Spectralomic Analysis. Appl. Opt..

[42] Paudel A., Raijada D., Rantanen J. (2015). Raman Spectroscopy in Pharmaceutical Product Design. Adv. Drug Deliv. Rev..

[43] Nagy B., Farkas A., Borbás E., Vass P., Nagy Z.K., Marosi G. (2018). Raman Spectroscopy for Process Analytical Technologies of Pharmaceutical Secondary Manufacturing. AAPS PharmSciTech.

[44] Tao Y., Bao J., Liu Q., Liu L., Zhu J. (2022). Application of Deep-Learning Algorithm Driven Intelligent Raman Spectroscopy Methodology to Quality Control in the Manufacturing Process of Guanxinning Tablets. Molecules.

[45] Karimi-Jafari M., Soto R., Albadarin A.B., Croker D., Walker G. (2021). In-Line Raman Spectroscopy and Chemometrics for Monitoring Cocrystallisation Using Hot Melt Extrusion. Int. J. Pharm..

[46] Simone E., Saleemi A.N., Nagy Z.K. (2014). Application of Quantitative Raman Spectroscopy for the Monitoring of Polymorphic Transformation in Crystallization Processes Using a Good Calibration Practice Procedure. Chem. Eng. Res. Des..

[47] Rehman G.U., Vetter T., Martin P.A. (2022). Design, Development, and Analysis of an Automated Sampling Loop for Online Monitoring of Chiral Crystallization. Org. Process. Res. Dev..

[48] Elderderi S., Wils L., Leman-Loubière C., Henry S., Byrne H.J., Chourpa I., Munnier E., Elbashir A.A., Boudesocque-Delaye L., Bonnier F. (2021). Comparison of Raman and Attenuated Total Reflectance (ATR) Infrared Spectroscopy for Water Quantification in Natural Deep Eutectic Solvent. Anal. Bioanal. Chem..

[49] Elderderi S., Wils L., Leman-Loubière C., Byrne H.J., Chourpa I., Enguehard-Gueiffier C., Munnier E., Elbashir A.A., Boudesocque-Delaye L., Bonnier F. (2021). In Situ Water Quantification in Natural Deep Eutectic Solvents Using Portable Raman Spectroscopy. Molecules.

[50] Elderderi S., Sacré P.-Y., Wils L., Chourpa I., Elbashir A.A., Hubert P., Byrne H.J., Boudesocque-Delaye L., Ziemons E., Bonnier F. (2022). Comparison of Vibrational Spectroscopic Techniques for Quantification of Water in Natural Deep Eutectic Solvents. Molecules.

[51] Makki A.A., Bonnier F., Respaud R., Chtara F., Tfayli A., Tauber C., Bertrand D., Byrne H.J., Mohammed E., Chourpa I. (2019). Qualitative and Quantitative Analysis of Therapeutic Solutions Using Raman and Infrared Spectroscopy. Spectrochim. Acta Part A Mol. Biomol. Spectrosc..

[52] Makki A.A., Massot V., Byrne H.J., Respaud R., Bertrand D., Mohammed E., Chourpa I., Bonnier F. (2021). Vibrational Spectroscopy for Discrimination and Quantification of Clinical Chemotherapeutic Preparations. Vib. Spectrosc..

[53] Belay N.F., Busche S., Manici V., Shaukat M., Arndt S.-O., Schmidt C. (2021). Evaluation of Transmission Raman Spectroscopy and NIR Hyperspectral Imaging for the Assessment of Content Uniformity in Solid Oral Dosage Forms. Eur. J. Pharm. Sci..

[54] Waffo Tchounga C.A., Sacré P.-Y., Ciza Hamuli P., Ngono Mballa R., De Bleye C., Ziemons E., Hubert P., Marini Djang’eing’a R. (2023). Prevalence of Poor Quality Ciprofloxacin and Metronidazole Tablets in Three Cities in Cameroon. Am. J. Trop. Med. Hyg..

[55] Cornel J., Lindenberg C., Mazzotti M. (2008). Quantitative Application of in Situ ATR-FTIR and Raman Spectroscopy in Crystallization Processes. Ind. Eng. Chem. Res.

[56] Byrne H.J., Bonnier F., McIntyre J., Parachalil D.R. (2020). Quantitative Analysis of Human Blood Serum Using Vibrational Spectroscopy. Clin. Spectrosc..

[57] Guleken Z., Jakubczyk P., Paja W., Pancerz K., Wosiak A., Yaylım İ., İnal Gültekin G., Tarhan N., Hakan M.T., Sönmez D. (2023). An Application of Raman Spectroscopy in Combination with Machine Learning to Determine Gastric Cancer Spectroscopy Marker. Comput. Methods Programs Biomed..

[58] Van Gheluwe L., Munnier E., Kichou H., Kemel K., Mahut F., Vayer M., Sinturel C., Byrne H.J., Yvergnaux F., Chourpa I. (2021). Confocal Raman Spectroscopic Imaging for Evaluation of Distribution of Nano-Formulated Hydrophobic Active Cosmetic Ingredients in Hydrophilic Films. Molecules.

[59] Lê L.M.M., Berge M., Tfayli A., Zhou J., Prognon P., Baillet-Guffroy A., Caudron E. (2018). Rapid Discrimination and Quantification Analysis of Five Antineoplastic Drugs in Aqueous Solutions Using Raman Spectroscopy. Eur. J. Pharm. Sci..

[60] Makki A.A., Elderderi S., Massot V., Respaud R., Byrne H., Tauber C., Bertrand D., Mohammed E., Chourpa I., Bonnier F. (2021). In Situ Analytical Quality Control of Chemotherapeutic Solutions in Infusion Bags by Raman Spectroscopy. Talanta.

[61] Bourget P., Amin A., Moriceau A., Cassard B., Vidal F., Clement R. (2012). La Spectroscopie Raman (SR): Un nouvel outil adapté au contrôle de qualité analytique des préparations injectables en milieu de soins. Comparaison de la SR aux techniques CLHP et UV/visible-IRTF appliquée à la classe des anthracyclines en cancérologie. Pathol. Biol..

[62] Afseth N.K., Kohler A. (2012). Extended Multiplicative Signal Correction in Vibrational Spectroscopy, a Tutorial. Chemom. Intell. Lab. Syst..

[63] Wartewig S. (2006). IR and Raman Spectroscopy: Fundamental Processing.

[64] Parachalil D.R., Brankin B., McIntyre J., Byrne H.J. (2018). Raman Spectroscopic Analysis of High Molecular Weight Proteins in Solution—Considerations for Sample Analysis and Data Pre-Processing. Analyst.

[65] Gautam R., Vanga S., Ariese F., Umapathy S. (2015). Review of Multidimensional Data Processing Approaches for Raman and Infrared Spectroscopy. EPJ Tech. Instrum..

[66] Greenacre M., Groenen P.J.F., Hastie T., D’Enza A.I., Markos A., Tuzhilina E. (2022). Principal Component Analysis. Nat. Rev. Methods Prim..

[67] Bonnier F., Byrne H.J. (2011). Understanding the Molecular Information Contained in Principal Component Analysis of Vibrational Spectra of Biological Systems. Analyst.

[68] Beattie J.R., Esmonde-White F.W.L. (2021). Exploration of Principal Component Analysis: Deriving Principal Component Analysis Visually Using Spectra. Appl. Spectrosc..

[69] Hibbert D.B. (2016). Vocabulary of Concepts and Terms in Chemometrics (IUPAC Recommendations 2016). Pure Appl. Chem..

[70] Guillot A.J., Martínez-Navarrete M., Garrigues T.M., Melero A. (2023). Skin Drug Delivery Using Lipid Vesicles: A Starting Guideline for Their Development. J. Control. Release.

[71] Larkin P. (2011). Infrared and Raman Spectroscopy: Principles and Spectral Interpretation.

[72] Mendelovici E., Frost R.L., Kloprogge T. (2000). Cryogenic Raman Spectroscopy of Glycerol. J. Raman Spectrosc..

[73] Parachalil D.R., Bruno C., Bonnier F., Blasco H., Chourpa I., Baker M.J., McIntyre J., Byrne H.J. (2019). Analysis of Bodily Fluids Using Vibrational Spectroscopy: A Direct Comparison of Raman Scattering and Infrared Absorption Techniques for the Case of Glucose in Blood Serum. Analyst.

[74] Orphanou C.-M., Walton-Williams L., Mountain H., Cassella J. (2015). The Detection and Discrimination of Human Body Fluids Using ATR FT-IR Spectroscopy. Forensic Sci. Int..

[75] Derenne A., Derfoufi K.-M., Cowper B., Delporte C., Goormaghtigh E. (2020). FTIR Spectroscopy as an Analytical Tool to Compare Glycosylation in Therapeutic Monoclonal Antibodies. Anal. Chim. Acta.

[76] Elderderi S., Hilali S., Wils L., Chourpa I., Soucé M., Clément-Larosière B., Elbashir A.A., Byrne H.J., Munnier E., Boudesocque-Delaye L. (2022). Monitoring Water Content in NADES Extracts from Spirulina Biomass by Means of ATR-IR Spectroscopy. Anal. Methods.

[77] Elderderi S., Leman-Loubière C., Wils L., Henry S., Bertrand D., Byrne H.J., Chourpa I., Enguehard-Gueiffier C., Munnier E., Elbashir A.A. (2020). ATR-IR Spectroscopy for Rapid Quantification of Water Content in Deep Eutectic Solvents. J. Mol. Liq..

[78] Tiernan H., Byrne B., Kazarian S.G. (2020). ATR-FTIR Spectroscopy and Spectroscopic Imaging for the Analysis of Biopharmaceuticals. Spectrochim. Acta Part A Mol. Biomol. Spectrosc..

[79] Czech K., Briggs J. (2020). Quantifying Ethanol Concentration in Hand Sanitizer via Automation. Appl. Noteb..

[80] Czech K., Briggs J., Technologies I. (2019). An Observation of Methanol in Ethanol Using Automated ATR Spectroscopy. Appl. Noteb..

[81] Sala A., Spalding K.E., Ashton K.M., Board R., Butler H.J., Dawson T.P., Harris D.A., Hughes C.S., Jenkins C.A., Jenkinson M.D. (2020). Rapid Analysis of Disease State in Liquid Human Serum Combining Infrared Spectroscopy and “Digital Drying”. J. Biophotonics.

[82] Bloomfield M., Andrews D., Loeffen P., Tombling C., York T., Matousek P. (2013). Non-Invasive Identification of Incoming Raw Pharmaceutical Materials Using Spatially Offset Raman Spectroscopy. J. Pharm. Biomed. Anal..

[83] Olds W.J., Sundarajoo S., Selby M., Cletus B., Fredericks P.M., Izake E.L. (2012). Noninvasive, Quantitative Analysis of Drug Mixtures in Containers Using Spatially Offset Raman Spectroscopy (SORS) and Multivariate Statistical Analysis. Appl. Spectrosc..

[84] Eliasson C., Matousek P. (2007). Noninvasive Authentication of Pharmaceutical Products through Packaging Using Spatially Offset Raman Spectroscopy. Anal. Chem..

[85] Ricci C., Eliasson C., Macleod N.A., Newton P.N., Matousek P., Kazarian S.G. (2007). Characterization of Genuine and Fake Artesunate Anti-Malarial Tablets Using Fourier Transform Infrared Imaging and Spatially Offset Raman Spectroscopy through Blister Packs. Anal. Bioanal. Chem..

[86] Rayyad A., Makki A.A., Chourpa I., Massot V., Bonnier F. (2022). Quantification of Clinical MAb Solutions Using Raman Spectroscopy: Macroscopic vs Microscopic Analysis. Talanta.

[87] De Beer T., Burggraeve A., Fonteyne M., Saerens L., Remon J.P., Vervaet C. (2011). Near Infrared and Raman Spectroscopy for the In-Process Monitoring of Pharmaceutical Production Processes. Int. J. Pharm..

[88] Hausman D.S., Cambron R.T., Sakr A. (2005). Application of Raman Spectroscopy for On-Line Monitoring of Low Dose Blend Uniformity. Int. J. Pharm..

[89] Vergote G.J., De Beer T.R.M., Vervaet C., Remon J.P., Baeyens W.R.G., Diericx N., Verpoort F. (2004). In-Line Monitoring of a Pharmaceutical Blending Process Using FT-Raman Spectroscopy. Eur. J. Pharm. Sci..

[90] Parachalil D.R., Bruno C., Bonnier F., Blasco H., Chourpa I., McIntyre J., Byrne H.J. (2019). Raman Spectroscopic Screening of High and Low Molecular Weight Fractions of Human Serum. Analyst.

[91] Bonnier F., Petitjean F., Baker M.J., Byrne H.J. (2014). Improved Protocols for Vibrational Spectroscopic Analysis of Body Fluids. J. Biophotonics.

[92] ICH, International Council for Harmonisation of Technical Requirements for Pharmaceuticals for Human Use Comitee ICH Harmonised Guideline: Validation of Analytical Procedures Q2(R2), Draft Version of March 2022. https://www.ema.europa.eu/en/ich-q2r2-validation-analytical-procedures-scientific-guideline.

